# Tracking the invasive hornet *Vespa velutina* in complex environments by means of a harmonic radar

**DOI:** 10.1038/s41598-021-91541-4

**Published:** 2021-06-09

**Authors:** Simone Lioy, Daniela Laurino, Riccardo Maggiora, Daniele Milanesio, Maurice Saccani, Peter J. Mazzoglio, Aulo Manino, Marco Porporato

**Affiliations:** 1 Department of Agricultural, Forest and Food Sciences, University of Turin, Grugliasco, Italy; 2 Department of Electronics and Telecommunications, Polytechnic University of Turin, Turin, Italy

**Keywords:** Conservation biology, Entomology, Animal migration, Invasive species

## Abstract

An innovative scanning harmonic radar has been recently developed for tracking insects in complex landscapes. This movable technology has been tested on an invasive hornet species (*Vespa velutina*) for detecting the position of their nests in the environment, in the framework of an early detection strategy. The new model of harmonic radar proved to be effective in tracking hornets either in open landscapes, hilly environments and areas characterised by the presence of more obstacles, such as woodlands and urban areas. Hornets were effectively tracked in complex landscapes for a mean tracking length of 96 ± 62 m with maximum values of ~ 300 m. The effectiveness of locating nests was 75% in new invasive outbreaks and 60% in highly density colonised areas. Furthermore, this technology could provide information on several aspects of insect’s ecology and biology. In this case, new insights were obtained about the mean foraging range of *V. velutina* (395 ± 208 m with a maximum value of 786 m) and flying features (ground speed), which was 6.66 ± 2.31 m s^−1^ for foraging individuals (hornets that are not carrying prey’s pellet) and 4.06 ± 1.34 m s^−1^ for homing individuals.

## Introduction

Understanding the movement of animal species is crucial for advancing our knowledge on several topics of species biology, ecology, social behaviour or ecophysiology, and generally for perceiving the role of individuals and populations within ecosystems^1^. This knowledge could be useful for planning conservation schemes for native species but also for understanding the movements and spread capabilities of invasive species in the introduced environments, to establish appropriate management strategies^2^. Approaches for tracking animal species vary according to taxa, species size and the environment in which they are tracked^1,3^. Due to their small dimension, insects are among the most difficult groups to be tracked, but they also represent a significant portion of terrestrial biodiversity that should be properly investigated^4,5^.

Since the last century, several technologies have been developed to follow the movement of insects in the environment, and therefore improve the knowledge already acquired with classical observational methods or mark-recapture studies^6^. Based on the presence or absence of batteries, tracking technologies can be roughly divided into two main categories: (1) active systems like radio telemetry^3^ or (2) passive systems that require the use of radar technologies^7,8^. The latter encompasses several variants such as vertical-looking radars for studying insect migration, scanning harmonic radars for low-altitude studies, and harmonic direction finders for low-range applications. Among radar techniques, scanning harmonic radars^9^ allow to understand the movement of insects in the environment for several hundred metres from the position of the radar, by applying light passive transponders (tags with a weight generally between 1 and 15 mg)^7,9^ on the insects that reflect the harmonic of the received radar signal, thereby minimising environmental interference (clutter). The narrow beam width of the radars used so far for tracking insects allowed to achieve a maximum detection range of 900 m in flat terrain conditions or flying arenas, although the reliable working range would usually be less (about 750 m)^7^. This technology has been tested on species of different characteristics and sizes, such as honey bees^10–12^, bumblebees^13,14^, butterflies^15^, moths and flies^7,8^, leading to new insights on species ecology and flying behaviour.

The scanning harmonic radar technique has been recently modified to overcome one of its main limiting factors, which is the operability in complex and hilly landscapes^16,17^. However, the increased beam width requested to operate in hilly areas generate a reduction of the overall detection range of the radar. Therefore, this modified scanning harmonic radar has been improved to further extend the tracking range up to about 500 m of radius from the radar position in flat terrain conditions^18^. This innovative scanning harmonic radar has been used with success for tracking the flight of *Vespa velutina* in Italy^18^, an invasive hornet species, which is colonising several countries of Europe and Asia, where it can generate impacts on multiple components, notoriously honey bee colonies, native insect communities, social concern related to the presence of nests in the environment and economic issues^19^.

Here we describe the performance of this new scanning harmonic radar in tracking insects in complex environments and non-controlled conditions, by analysing its application in the detection of nests of *V. velutina* as a case study. Performances of the harmonic radar are evaluated in terms of:

1. success in tracking *V. velutina* workers from the apiaries, where hornets are hunting honey bees, to their nests;
2. length of the tracks in relation to the environmental characteristics in which the harmonic radar is operating;
3. length of the tracks recorded with the harmonic radar in relation to a traditional tracking technique, which is the visual tracking and triangulation of flying directions^20^.

Moreover, we highlight how the harmonic radar tracking could be used to deepening the knowledge on several aspects of insect’s ecology and biology in natural and non-controlled conditions: in this case, the flying characteristics (ground speed) and the distances of *V. velutina* colonies from apiaries where hornets were preying on honey bees (foraging range).

Hornets were tracked in Italy in nine localities of Liguria (Supplementary Table S1) with different characteristics in terms of land cover (open terrains, urban areas, and woodlands), road density, elevation, slope degree and *V. velutina* density. Four of these localities were new invasive outbreaks where the species was present at low densities and the predation pressure on honey bee colonies was restrained, while the other localities were inside the area that had been colonised by the species since 2014^21^. Hornets have been tagged and tracked with the harmonic radar and the transponders designed, developed, and previously described by the authors^18^ (see “Methods” for detailed insights on the tracking procedure).

## Results and discussion

### Tracking success

**Figure 1:**
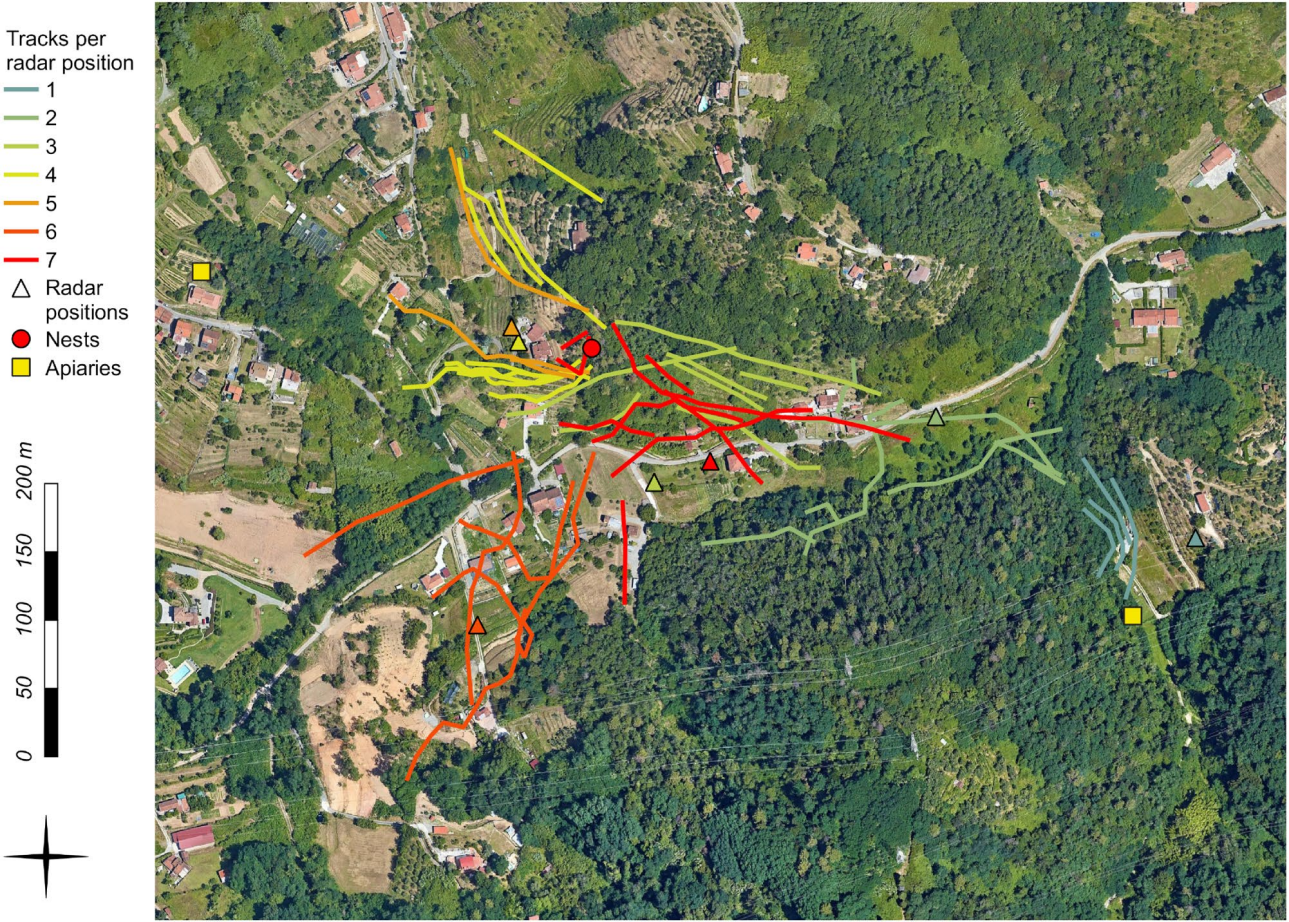
Example of *V. velutina* tracking session with the harmonic radar in the invasive outbreak of Arcola (La Spezia, Italy). *V. velutina* workers were tagged (*n* = 14) in the apiary close to the first radar position (the easternmost apiary on the map). Subsequently, the harmonic radar was moved accordingly to the flying direction of hornets in other six positions (triangles). Different colours highlight the recorded tracks (*n* = 46) in relation to each radar position. The red dot indicates the position of the discovered nest of *V. velutina*. Background map by Google Maps (maps.google.com).

The harmonic radar tracking allowed us to discover the position of several *V. velutina* nests (*n* = 11) in six of the nine localities where hornets were tracked, with an efficiency of 75% in outbreaks and 60% in high-density colonised areas (Supplementary Table S1). The result of the tracking session in the outbreak of Arcola (La Spezia district) is displayed as an example (Fig. 1), while the results from the other localities are available as supplementary information (Supplementary Figs. S1–S11). The efficiency in locating nests is similar (or even slightly higher when considering outbreaks) to the results obtained with another tracking technique (radio telemetry) in other European countries, where the authors achieved a 63% of success rate in nest detection^22^. The overall lower efficiency that we recorded in high-density colonised areas than outbreaks is probably related to the presence of multiple nests, which implies that tagged hornets may fly towards different colonies. This aspect could complicate the identification of the main flying paths and consequently decrease the probability of nest detection.

Nests of *V. velutina* detected with the harmonic radar tracking were located at a mean distance of 395 ± 208 m (*M* ± *SD*, *n* = 10) from the apiaries where hornets were preying on honey bees (min = 72 m, max = 786 m, Supplementary Table S2). These data are consistent with previous information on the possible foraging range of *V. velutina*, which is probably in a radius around the nest of 500–700 m^23,24^ and up to a few kilometres (e.g. a nest was detected near Bordeaux at 1.33 km from the foraging area^22^; in the present study, a tagged hornet was reported by a beekeeper when foraging in an apiary located at 2.3 km from the detected nest). When nests were detected, a mean of 35 ± 20 hornets had been tagged (overall tagged hornets in the nine localities: *n* = 657). In these cases, the harmonic radar was used for 11 ± 4 h (overall hours of radar operation time: *n* = 190) from a mean of 3 ± 2 positions (overall radar positions: *n* = 47), with a maximum of seven positions for the same tracking session (Supplementary Table S2). The movement of the radar was necessary to overcome physical obstacles that could limit the transmission of the signal, or for resuming flying paths of hornets that flew out of the detection range. To reduce handling time or prevent this issue, the scanning harmonic radar might be easily mounted on a van or a cross-country vehicle.

Environmental characteristics of the localities are different in terms of land cover, elevation above sea level, slope gradient and road density. Outbreak areas were generally characterised by a prevalence of urbanised or woodland landscapes, while localities inside the colonised range were generally formed by open terrains (Supplementary Fig. S12; Supplementary Table S3). Study areas where the harmonic radar tracking did not allow to detect nest positions were characterised by: (1) highly urbanised areas, in which some difficulties may arise with the presence of private proprieties that could limit the movement of the radar and the inspection of the areas at the end of the recorded tracks (Supplementary Fig. S6), (2) woodland landscapes with low values in road densities, thus limiting the possibilities of radar’s movement (Supplementary Fig. S7) and (3) areas with steep slopes (Supplementary Fig. S9).

### Length of the tracks

**Figure 2:**
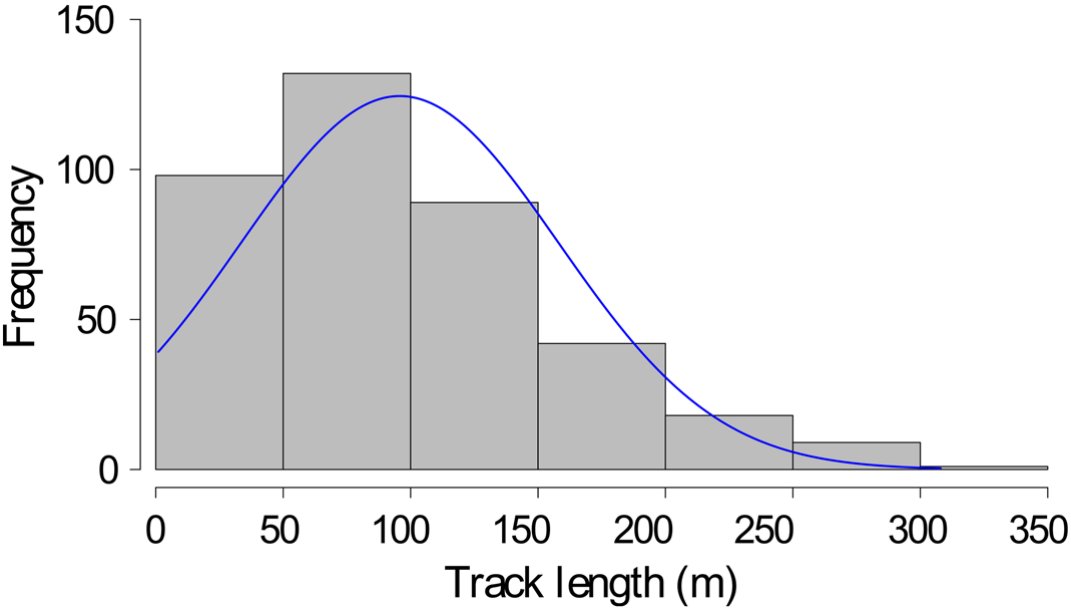
Frequency histogram of the length of the tracks of *V. velutina* workers tracked with the harmonic radar. Overall tracks recorded in the nine localities (*n* = 389) and divided by length intervals of 50 m. The line represents the fitting of a normal distribution to the length of the tracks.

In the nine localities, hornets were tracked through the harmonic radar for an overall length of 37 km, by recording 2580 fixes (positions of the tagged hornets) that allowed the reconstruction of 389 tracks of single hornets (see “Methods” for further insights). The mean length per track was 96 ± 62 m with a maximum value of 308 m (Fig. 2). Mean tracking length was different just in the case of Dolceacqua (67.7 ± 52.6 m), where tracks were significantly shorter (Supplementary Figs. S13-S14) than tracks in Arcola (106.4 ± 62.6 m, *P* = 0.023), Finale Ligure (109.5 ± 65.1 m, *P* = 0.008) and Calvo in Ventimiglia (112.5 ± 66.7 m, *P* < 0.001) (Kruskal–Wallis test: *H* = 30.25, *df* = 8, *P* < 0.001; differences between groups evaluated with the Dunn test with Bonferroni correction). This means that, overall, the performance in tracking flying hornets were similar among study areas.

**Figure 3:**
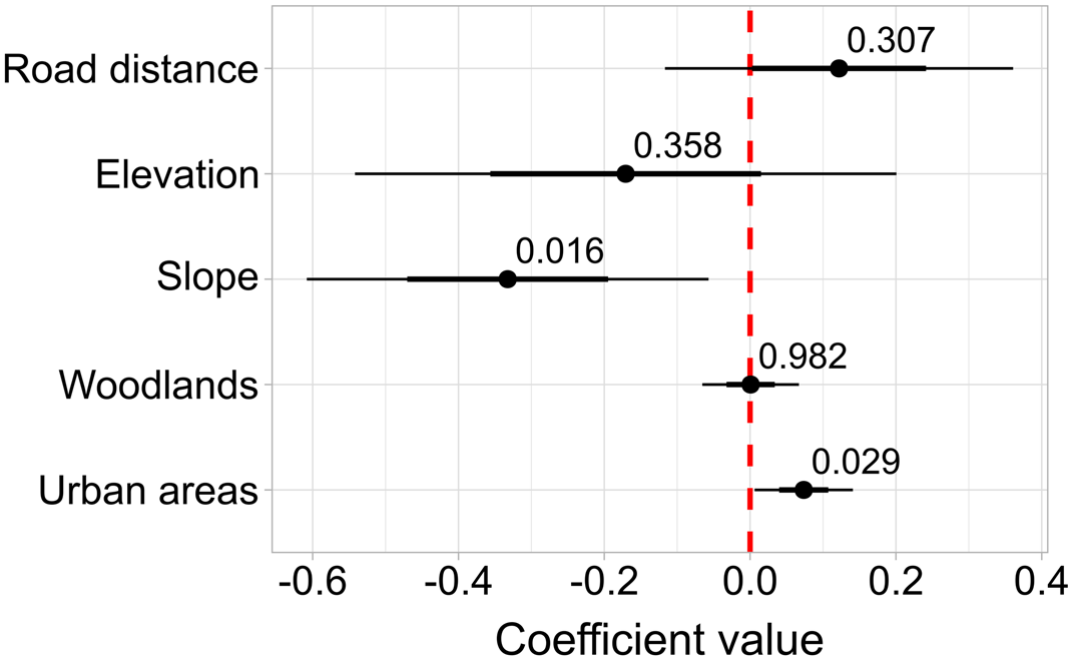
Influence of environmental characteristics on the length of the tracks of *V. velutina* workers tracked with the harmonic radar. The coefficient plot of the GLMM analysis indicates that the slope degree is the only significant variable that negatively affects the length of the tracks recorded with the harmonic radar, while land cover, elevation and road distance have no negative effects. The dots depict the modelled effects (*P* value is reported), inner bars the CI at 50% and outer bars the CI at 95% (see also Supplementary Table S4; Supplementary Fig. S15).

A GLMM analysis on environmental characteristics indicate that the slope gradient was the main environmental variable that affected tracking lengths with the harmonic radar (Fig. 3, Supplementary Fig. S15; Supplementary Table S4). Despite this negative influence, we were able to track hornets in the worst scenario in terms of slope degree (25.4 ± 7.6° in Latte of Ventimiglia) for a mean distance of 107 ± 73 m per track, with extreme values up to 298 m. On the contrary, land cover does not influence tracking performances negatively since hornets were effectively tracked in open terrains as well as in areas characterised by the presence of more obstacles, such as woodlands or urban areas (Fig. 3, Supplementary Table S4).

## Comparison of tracking techniques

**Figure 4:**
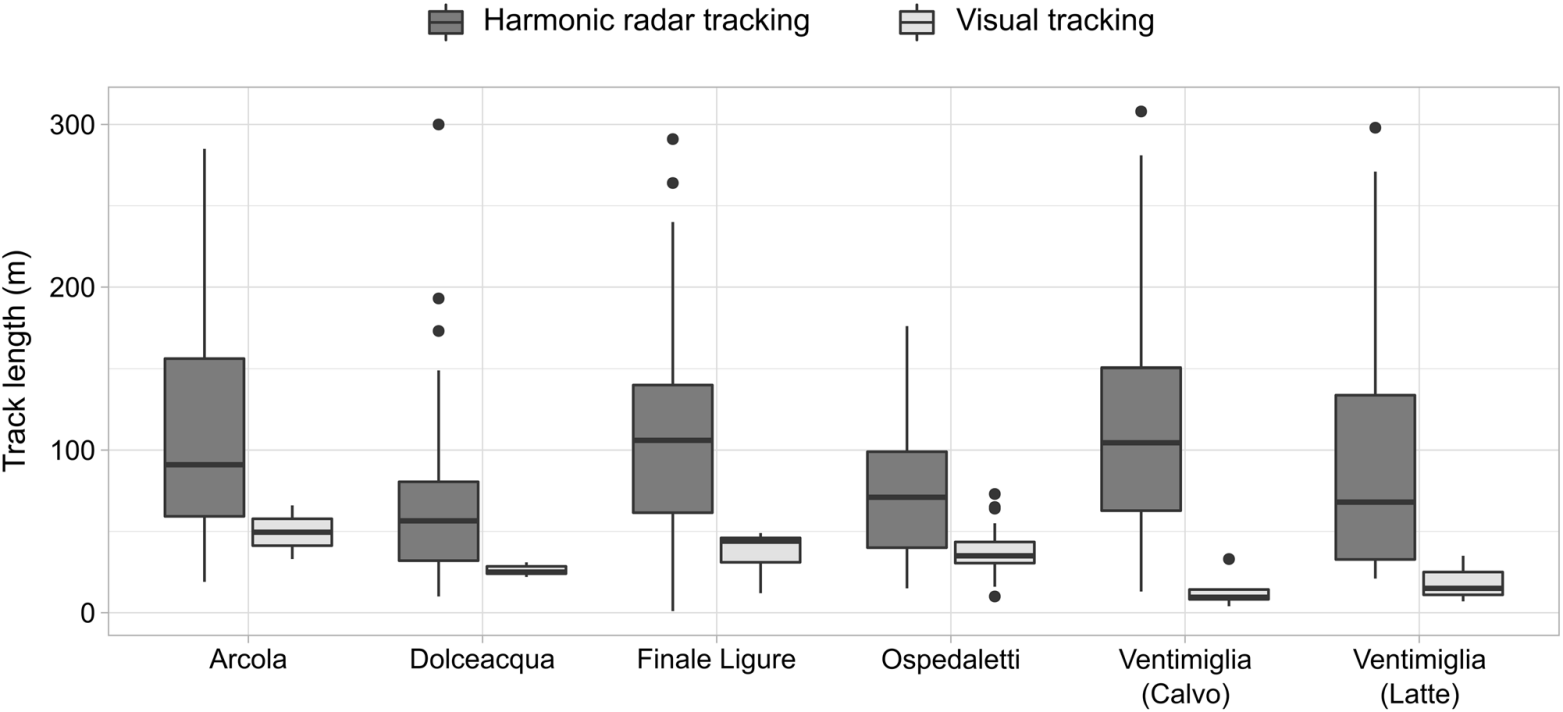
Comparison between techniques for tracking *V. velutina*. Boxplot of the length of the tracks recorded with the harmonic radar (dark grey) and the length of the tracks recorded with the customary technique of visual tracking (light grey). Sample size per locality is: Arcola (harmonic radar tracking: *n*_hr_ = 46; visual tracking: *n*_v_ = 2), Dolceacqua (*n*_hr_ = 50, *n*_v_ = 7), Finale Ligure (*n*_hr_ = 43, *n*_v_ = 25), Ospedaletti (*n*_hr_ = 25, *n*_v_ = 19), Calvo of Ventimiglia (*n*_hr_ = 92, *n*_v_ = 10), Latte of Ventimiglia (*n*_hr_ = 40, *n*_v_ = 3). Horizontal line represents the median and points depict outlier values.

The performances of the harmonic radar tracking, in terms of length of the tracks, were higher than the performance of the technique customary used for following hornets, which is the visual tracking and triangulation of flying directions (Fig. 4). In the six localities where the tracking length was compared, the harmonic radar allowed to track hornets for a mean length of 98 ± 65 m per track (number of tracks: *n* = 296), while visual tracking reached a mean length of 32 ± 16 m (*n* = 66), with a significant difference between the two methods (GLMM: *β*_visual_tracking_ = -1.07, *SE* = 0.09, *P* < 0.001; null model comparison: *χ*^*2*^ = 98.52, *df* = 1, *P* < 0.001). Nevertheless, other aspects should be taken into account when comparing tracking techniques, such as the effectiveness in detecting nests, the required time and the associated costs. The first two factors depend on multiple variables, including landscape characteristics, presence of obstacles and experience of the operators, and only a blind experiment in the same study area may allow to rigorously compare advantages and disadvantages of different techniques. Since this was not the case, here we discuss the time for locating nests, the associated costs, and advantages/limits of the main tracking techniques adopted for locating nests of *V. velutina* in relation to our findings and to the information available from literature.

The results from the control strategy established in the Balearic Islands^25^ allow delineating some hypothesis concerning the time for locating nests for the visual tracking technique, notwithstanding different environmental characteristics of the study areas may act as confounders. In their invasive scenario of Majorca, the authors were able to detect and remove 30 nests of *V. velutina* in the years 2015–2017 by visual tracking and triangulation of flying directions; this activity required a mean of 19.2 ± 18.9 working days per nest (min = 0 days, max = 63 days). With the harmonic radar tracking, we were able to locate nests in Italy with a mean of 2.5 ± 1.0 working days (min = 2 days, max = 5 days) and 11 ± 4 h of effective radar use. In terms of time for detecting nests, a higher performance in the use of tracking technologies than visual tracking has also been highlighted for the radio-tracking technique^22^, with which the authors were able to detect *V. velutina* nests in 92 ± 37 min. However, an aspect that should be taken into account with the latter technique is the weight of the tags, which could range from 150 to 312 mg (10–21 times heavier than the tag used for the harmonic radar technique) or more. This heavier weight might affect the flying capacities of the hornets, particularly in spring or early summer when the weight of *V. velutina* workers is lower^26^.

Another aspect that should be considered when comparing tracking techniques is the associated costs (personnel and equipment). All the above-mentioned techniques could be easily performed by a team of two trained people, despite the visual tracking technique requires, in general, a higher number of working days and therefore higher personnel costs. Concerning technological techniques, initial equipment costs are higher for the harmonic radar tracking (approximately 100 k euro per unit). On the other hand, radio-tracking has lower initial equipment costs than harmonic radar tracking, but a higher cost related to the tags for tracking insects.

Therefore, selecting a tracking technique for locating *V. velutina* nests requires an analysis of advantages and limits in relation to the available resources, the characteristics of the landscapes, and the urgency that is required to find the nests in relation to the invasive scenario (Table 1). The use of thermal imaging cameras^27^, in association to the selected tracking technique, may also facilitate spotting the location of the nest once the area of presence has been defined.

**Table 1:** Advantages, disadvantages, and costs of different tracking techniques.

	Harmonic radar tracking	Radio tracking	Visual tracking
**Applicability of the method for detecting *V. velutina* nests**			
Effectiveness in detecting *V. velutina* nests	+	+	+
Time for nest detection	+	+	−
Infield movement simplicity	−	+	+
Applicability in relation to the weight of the hornets	+	−	+
**Personnel and equipment costs**			
Initial equipment costs	−	+	+
Tag costs	+	−	+
Personnel costs in relation to the time for detecting nests	+	+	−
**Exportability for other applications**			
Exportability to other insect species of different weight	+	−	+
Range of ecological information that can be discovered	+	−	−

Positive (+) and negative (−) aspects of the different tracking techniques are reported, in relation to the: (1) applicability of the method for detecting *V. velutina* nests; (2) costs (personnel and equipment) for implementing the tracking technique; (3) exportability to other applications (collection of ecological data and applicability to other insects).

### *Vespa velutina* flying speed

**Figure 5:**
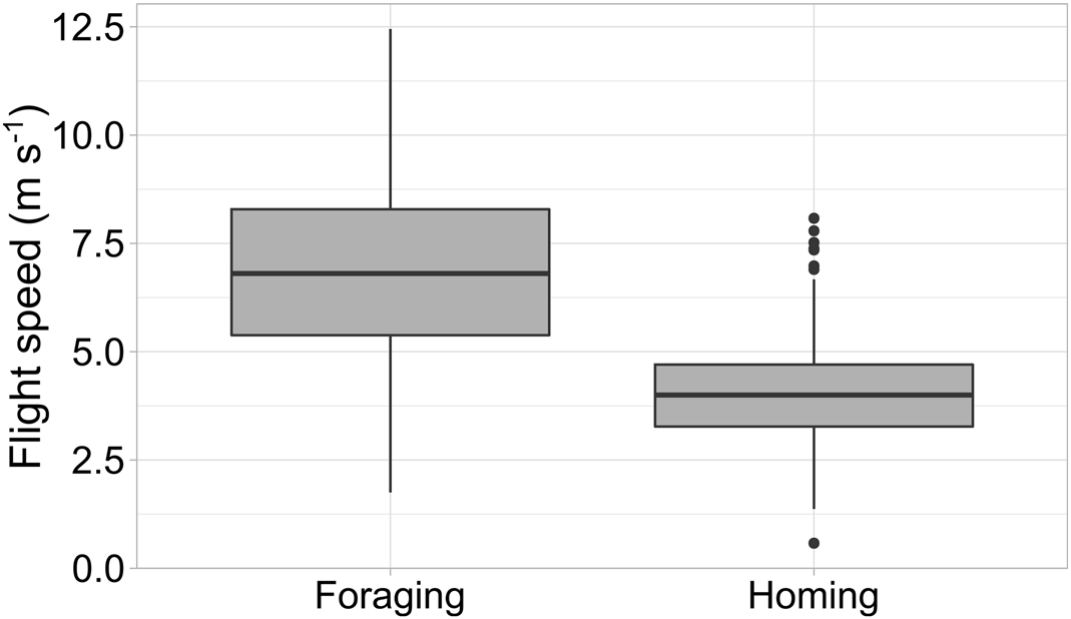
Flight speed of *V. velutina* workers. Foraging (*n* = 130) are hornets flying from the nest to the apiary for hunting honey bees, homing (*n* = 186) are hornets flying back to the nest from the apiary. Horizontal line represents the median and points depict outlier values.

In the three localities of La Spezia district, foraging hornets were flying at a mean ground speed of 6.66 ± 2.31 m s^−1^ (*n* = 130) whereas homing hornets had a mean ground speed of 4.06 ± 1.34 m s^−1^ (*n* = 186; Fig. 5). Flying speed is significantly different between the two groups (Wilcoxon rank-sum test: *W* = 19,830, *P* < 0.001), and values are consistent with previous findings for honey bees (3.6–5.6 m s^−1^)^11^, bumblebees (3.0–15.7 m s^−1^)^13^ and hornets (5.9 m s^−1^)^28^. Moreover, good weather conditions were present while tracking hornets in La Spezia, with mean wind speed values ranging between 1.4 and 2.4 m s^−1^ (*M* = 1.8, *SD* = 0.3).

The difference between foraging and homing hornets is probably related to the weight (part of the body of honey bees preyed by the hornets) that homing hornets are carrying to their nests for feeding the brood, but could also be connected to the energy expenditure that hornets encountered during their predation activity. Moreover, since the weight of *V. velutina* workers may change considerably from spring to autumn^26^, it is possible that mean flying speed could change accordingly. Here we estimated ground flying speed of hornets tracked in autumn (from the 11th of September to the 3rd of October), thus hornets in spring and early summer may show a lower flying speed than hornets with a higher body mass.

Finally, these findings show that the flying characteristics of *V. velutina* in natural conditions are considerably different from the values recorded in laboratory conditions, e.g. with flight mill experiments (1.6 m s^−1^)^29^, underlining the necessity of instruments for the direct study of flying insects in their natural environments and in non-controlled conditions.

### Implications

Our findings highlight the performance of a recent scanning harmonic radar technology that has been designed and developed for tracking flying insects in natural and complex environments^16–18^. This technology has been used in the framework of a management strategy developed to contain the spread of an invasive hornet species in Italy^19,30^, leading to the detection of *V. velutina* nests either in low-density invasive outbreaks or high-density colonised areas. We were able to operate with the same performances in open terrains as well as areas characterised by the presence of more obstacles, such as woodlands and urban areas, finding that the only limiting factor is represented by the slope degree, due to the intrinsic characteristics of scanning harmonic radars (beam width). However, also in complex environments characterised by a high degree of slopes (25.4 ± 7.6°), hornets were effectively tracked up to about 300 m of length per single tracks.

These findings highlight the potentialities posed by this recent scanning harmonic radar in tracking and studying the movement of insects in the ecosystems. This radar can be used to extend, in complex natural environments, research works already implemented in flying arenas or flat environments, for studying for example the foraging range of flying insects and their interactions with the ecosystems^13^, flying behaviours^11,12^, dispersal capabilities^14^ or evolutionary aspects^15^. At the same time, it could be used for the management of invasive insect species with similar nesting behaviour, such as several other wasp species^31^, in the framework of an early warning and rapid response strategy against biological invasions.

## Methods

### Study areas

The technique of harmonic radar tracking has been applied in nine different localities of Liguria (Italy), in the framework of the control activities developed to contain the spread of *V. velutina* in this region^19,21,30^. Four of these study areas (Ameglia, Arcola, Riccò del Golfo in La Spezia district and Finale Ligure in Savona district) were new invasive outbreaks characterised by a low nest density of *V. velutina* and low predation pressure on honey bee colonies. The other five study areas of Imperia district (Camporosso, Dolceacqua, Ospedaletti, and the two villages of Calvo and Latte in the municipality of Ventimiglia) were located inside the colonised range of the species^21^, and were characterised by a high nest density and an intensive predation pressure on honey bee colonies (Supplementary Table S1).

### Harmonic radar tracking

**Figure 6:**
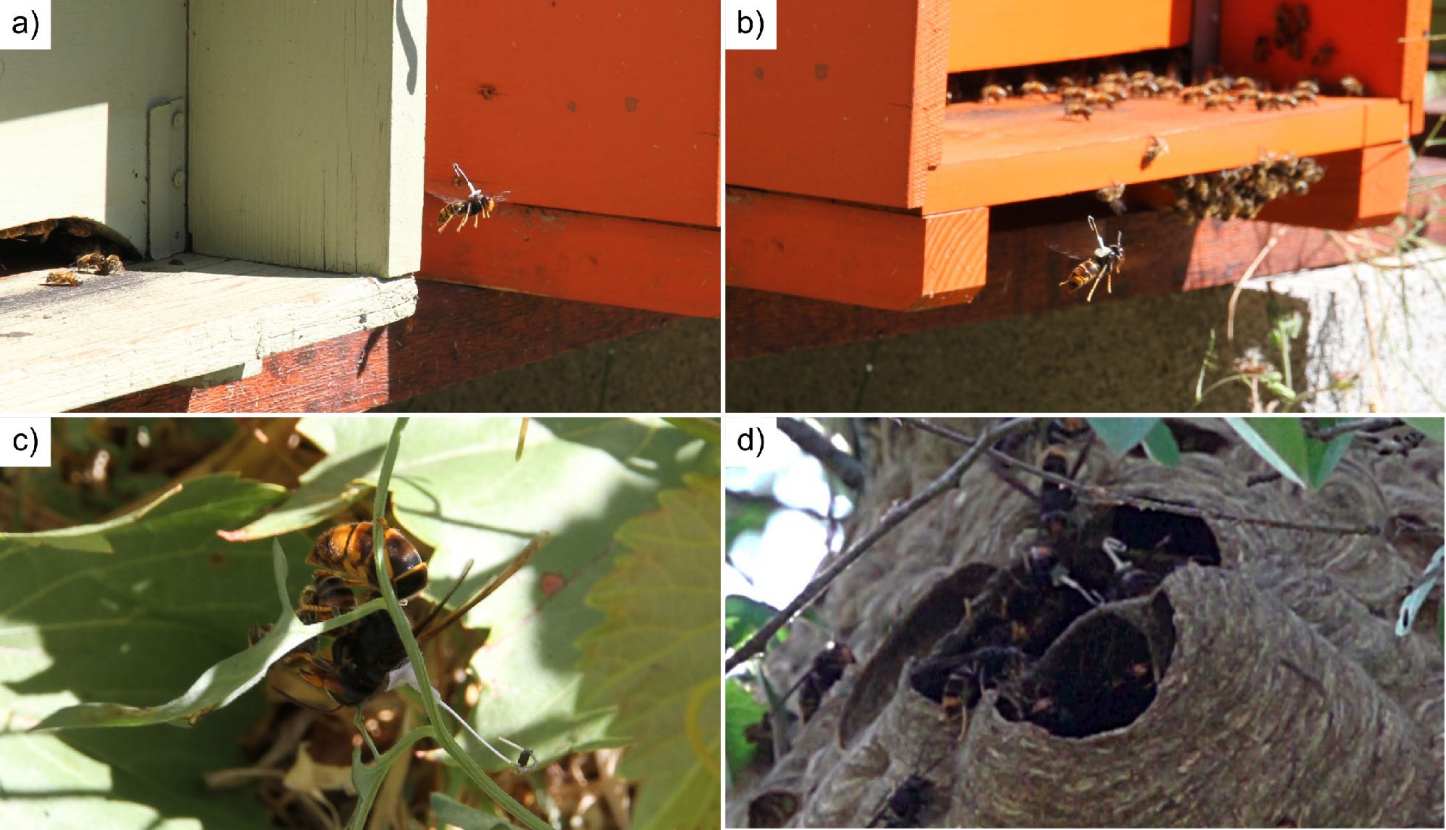
Tagged hornets performing their usual predatory behaviour. Tagged individuals of *V. velutina* hovering in front of honey bee colonies for preying on forager bees (**a**,**b**). A tagged hornet that is disjointing a honey bee for gathering the thorax (most energetic part of its prey), that will be brought back to the nest for feeding the brood (**c**). Two tagged hornets in proximity of the entrance hole of the nest (**d**).

The harmonic radar and the tags that have been used for tracking the flight of *V. velutina* were designed and developed ad-hoc for following insects in complex environments; their technical and innovative characteristics have been previously described by the authors^18^. At the beginning of a new tracking session, worker hornets are trapped, usually in apiaries while preying on honey bees, and the transponders are attached on their thorax using an orthodontic glue, without anesthetising the insects. Subsequently, hornets are released from the tagging location and are immediately able to resume their activity, such as flying and preying on honey bees (Fig. 6). The whole tagging procedure requires less than one minute per hornet. Tag weight (15 mg) is approximately 4–7% of the weight of *V. velutina* workers (mean worker’s weight changes over the season between 189 and 386 mg)^26^. Moreover, the tag is 3–4 times lighter than the weight of prey’s pellet generally transported to the nest by this species. This information, together with multiple observations of tagged hornets in apiaries and the results achieved by other authors with a radio-tracking experiment (in which it was found that hornets equipped with a tag of weight lesser than 80% of their body weight are considered good flyers)^22^, suggest that the tags used in this study do not affect the behaviour and the flying abilities of *V. velutina*.

The harmonic radar records independently all the tracks of flying hornets that are inside its detection range. The real-time analysis of the recorded tracks allows understanding the main flying directions. If the nest of *V. velutina* is located outside of the maximum detection range of the radar (about 500 m in flat terrain)^18^ or behind physical obstacles, the harmonic radar is moved according to the flying directions of the hornets. The presence of a diffused road network, as in many of our study areas, facilitated the movement of the radar from one position to another. This operation is repeated until the position of the nest is determined. The area where the nest is located is generally highlighted by the presence of several tracks that converge or begin from the same site. The visual inspection of the area permits the exact detection of the position of the nest. In several cases, tagged hornets were visually observed on the surface of the nests (Fig. 6d).

The total number of tagged hornets was recorded for each tracking session, together with the radar operation time, the number of radar movements per session, the number of detected nests per session and the minimum distance between the nests and the apiaries where hornets were hunting honey bees (Supplementary Table S2). Hornets were trapped with standard entomological procedures for trapping insects, and experiments were conducted ethically since no hornets were killed, injured, or kept captive after being tagged.

### Tracking lengths and environmental characteristics

The main parameter selected for estimating the performance of the harmonic radar in tracking *V. velutina* in different natural and complex environments is the length of the tracks of tagged insects. To obtain this parameter, fixes (hornets detected by the harmonic radar at each radar’s rotation) were extracted for each tracking session and uploaded on a GIS software^32^. Afterwards, consecutive fixes of the same track were connected with the shortest line, so to obtain hornet tracks and calculate their length. The advanced radar analyses used for processing the received signals^18^ allow discriminating the true fixes (position of the hornet) from clutter (reflected signals received from objects in the landscape). However, the presence of obstacles may generate gaps in the received signals (e.g. when a hornet is temporarily flying behind an obstacle such as a house), but these gaps were rare and never occurred for long periods of time. In these cases, if fixes were not clearly recognizable to a track of the same hornet, these were excluded from the analysis. The exclusion of the tracks was performed also in the rare cases during which the presence of multiple tagged hornets did not allow a clear identification of the tracks.

The length of the tracks in each fix position (*n* = 2580) was modelled with a GLMM (see “Data analysis”) to evaluate the effect of environmental features (land cover, elevation above sea level, slope gradient, road density). The land cover layer was obtained through a photo interpretation of satellite images (in a buffer area of 100 m around the minimum convex polygon that encompass all the tracks in each locality) and classification in three macro-levels: open terrains (landscapes predominantly characterised by open areas, such as fields), urban areas (matrices formed by buildings/roads) and woodlands (matrices formed by forests). Elevation above sea level and slope degree were obtained by a digital elevation model (resolution of 20 m).

### Visual tracking of flying hornets

The length of the tracks recorded by the harmonic radar was compared with the length of the tracks recorded when adopting a customary technique for tracking insects, such as the visual tracking and triangulation of flying directions^20,25^. In six of the nine localities where the harmonic radar tracking has been applied (Fig. 4), an operator was waiting near a honey bee colony till one *V. velutina* worker caught a honey bee. Subsequently, after the hornet disjoined the most energetic parts of its prey (the thorax)^33^, the operator visually tracked the flight of the hornet when flying back to its nest, using a binocular and by recording with a GPS the position where the hornet disappeared from view. In some cases (*n* = 4), common flying routes were identified, and we were able to resume the visual tracking with other hornets from the previous disappearance position. Finally, GPS positions were uploaded on a GIS software to calculate the length of the tracks with this technique.

In this study, the visual tracking technique has not been implemented systematically for nest detection, therefore the two approaches are compared only by evaluating the recorded length of the tracks. The effectiveness in locating nests, the required time and the associated costs are discussed in the framework of previous studies for tracking *V. velutina*, taking into account advantages and limits of the different techniques^20,22,25^.

### Estimation of *V. velutina* ground flying speed

Harmonic radar tracking allows estimating the ground flying speed of *V. velutina*, by analysing the distance between each recorded position at consecutive radar rotations. Giving that the time of each radar rotation is fixed (3 s), it is possible to estimate the hornet’s speed between each detection^8^.

The ground flying speed of *V. velutina* has been estimated in the three localities of La Spezia district, due to the availability of a subsample of clear tracks with consecutive detections per each rotation of the radar and good weather conditions. Furthermore, based on their direction, tracks were classified in homing tracks (*H)*, which belong to hornets flying from the apiary to the nest, and foraging tracks (*F*), which belong to hornets flying towards the apiary for hunting honey bees. Data on wind speed and direction were obtained from weather stations close to the study areas.

### Data analysis

Data analyses were performed with the software R^34^. Environmental characteristics of the localities were analysed with a Principal Component Analysis (PCA; package *factoextra*), to understand affinities between study areas and correlations between the considered variables. The length of the tracks between localities recorded with the harmonic radar was compared with the Kruskal–Wallis and the Dunn tests with Bonferroni correction, while the flying speed between foraging and homing hornets was compared with the Wilcoxon rank-sum test (two-tailed).

Generalized linear mixed models (GLMM; package *lme4*) with gamma distribution and log link function were used to assess (1) the influence of environmental variables on the length of the tracks and (2) compare tracking methods between study areas. In the first case, a random slope model has been implemented, by defining the locality and the slope degree as random effects (uncorrelated). In the second case, a standard random intercept model has been implemented, by selecting the locality as random effect. In both cases, continuous variables were standardized, and multi-collinearity of environmental variables was taken into account by calculating the Variance Inflation Factor (VIF). This was 1.5 for elevation and slope degree, and 1.0 for road density.

## Supplementary Information

Supplementary Information 1.

## Data Availability

The data that support the findings of this study are available from the corresponding author upon justified request.
