# Supplementary material for: Tracking the invasive hornet Vespa velutina in complex environments by means of a harmonic radar
Source: Sci Rep. 2021 Jun 9;11:12143. doi: 10.1038/s41598-021-91541-4 (PMC8190064; doi:10.1038/s41598-021-91541-4)
Supplement: Supplementary file 1 — Supplementary Information 1. [file 41598_2021_91541_MOESM1_ESM.pdf]

Tracking the invasive hornet *Vespa velutina* in complex environments by means of a harmonic radar

Simone Lioy[1], Daniela Laurino[1], Riccardo Maggiora[2], Daniele Milanesio[2], Maurice Saccani[2], Peter J. Mazzoglio[1], Aulo Manino[1] & Marco Porporato[1]

[1] Department of Agricultural, Forest and Food Sciences, University of Turin, Grugliasco, Italy
[2] Department of Electronics and Telecommunications, Polytechnic University of Turin, Turin, Italy

Supplementary information

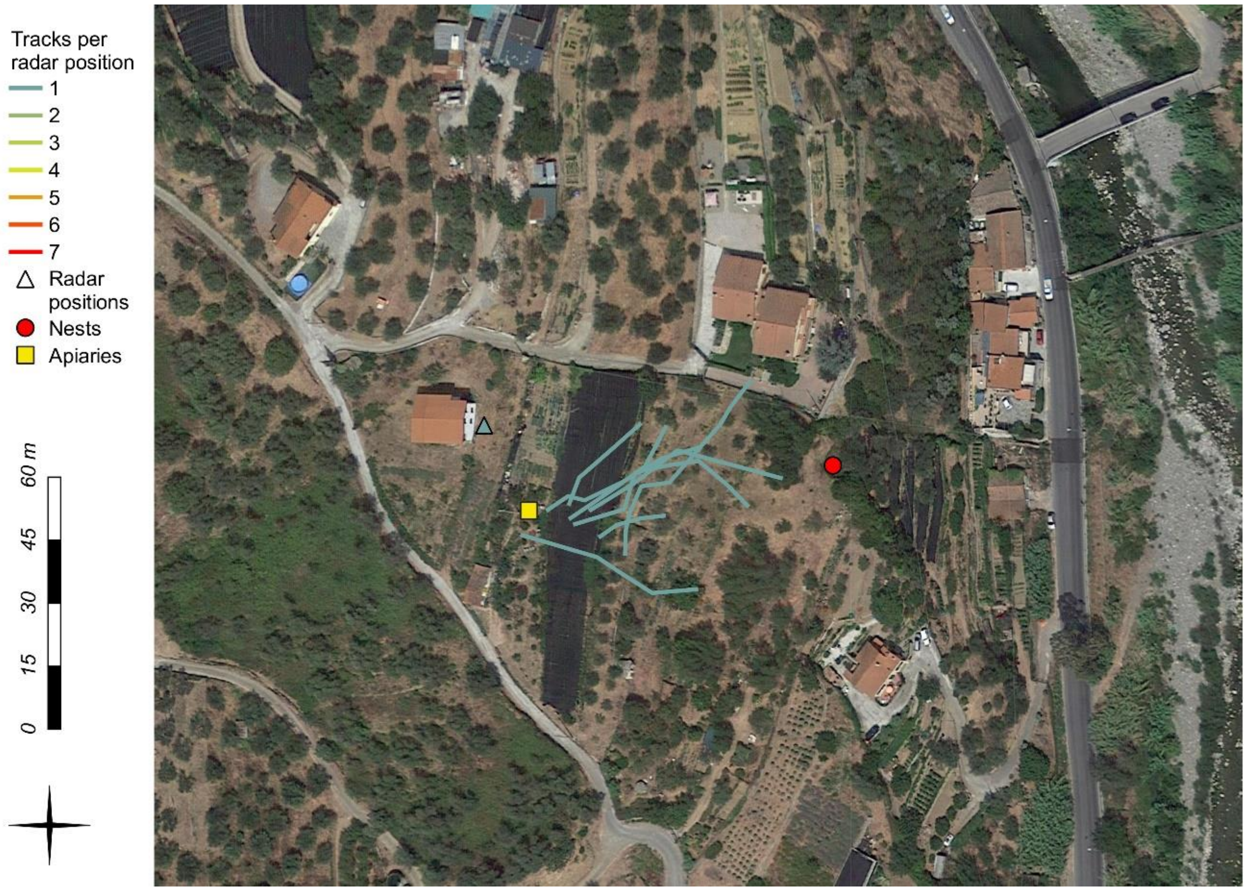

Supplementary Fig. S1 Tracking session in Dolceacqua (31 August - 1 September 2017). Hornets were tagged in the apiary (yellow square), tracked with the harmonic radar from one position (triangle) until nest position was discovered (red dot). Background map by Google Maps (maps.google.com).

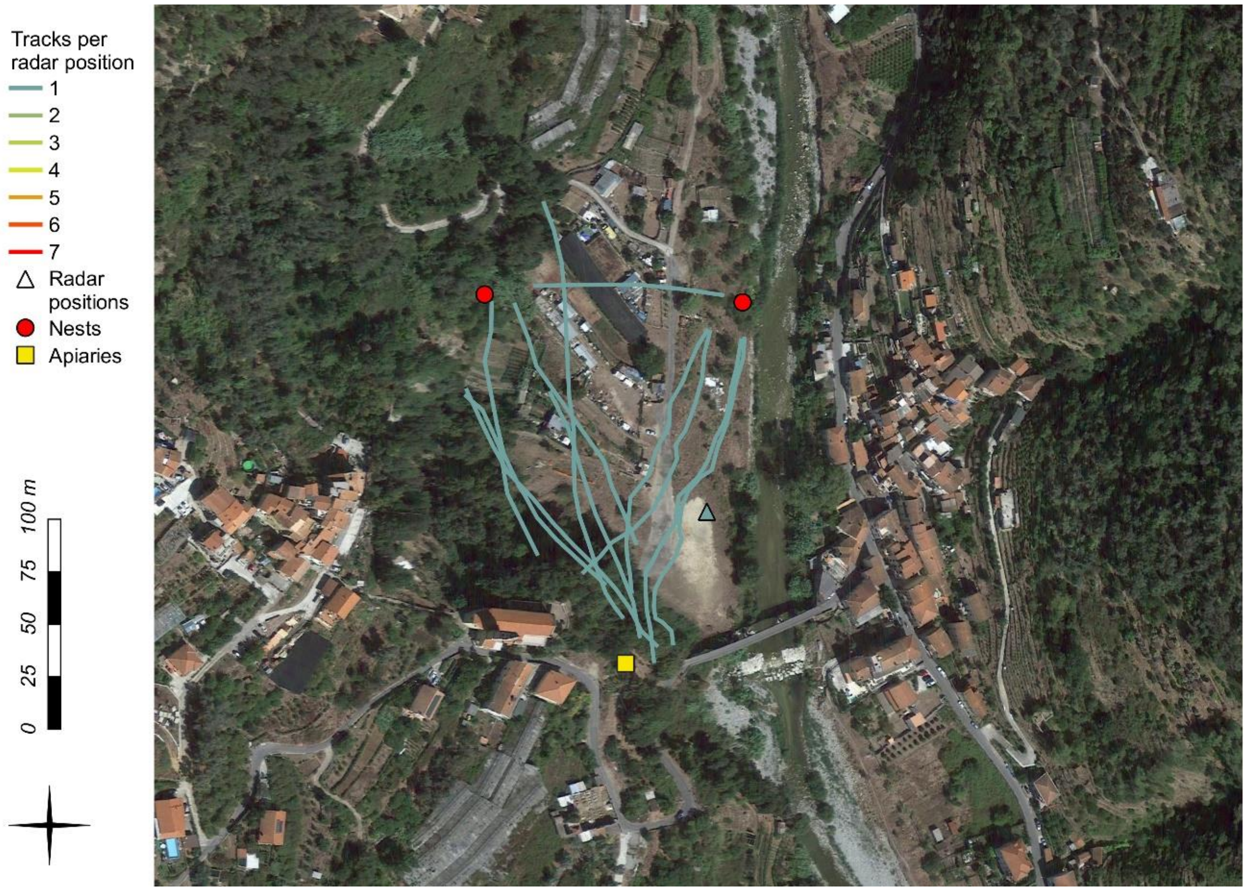

Supplementary Fig. S2 Tracking session in the village of Calvo in Ventimiglia (26-27 October 2017). Hornets were tagged in the apiary (yellow square), tracked with the harmonic radar from one position (triangle) until the position of two nests was discovered (red dots). Background map by Google Maps (maps.google.com).

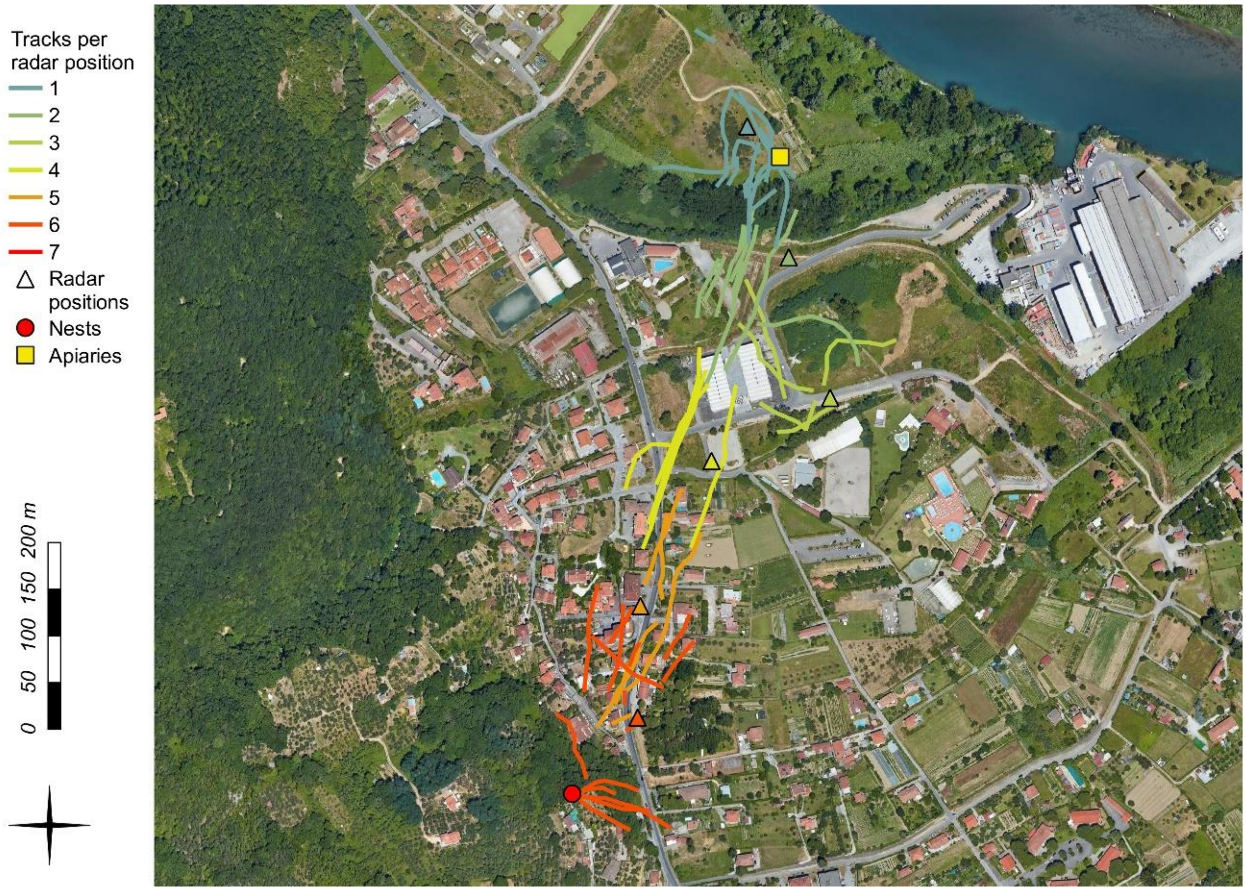

Supplementary Fig. S3 Tracking session in the outbreak of Ameglia (18-19 September 2018). Hornets were tagged in the apiary (yellow square), tracked with the harmonic radar from six positions (triangles) until nest position was discovered (red dot). Background map by Google Maps (maps.google.com).

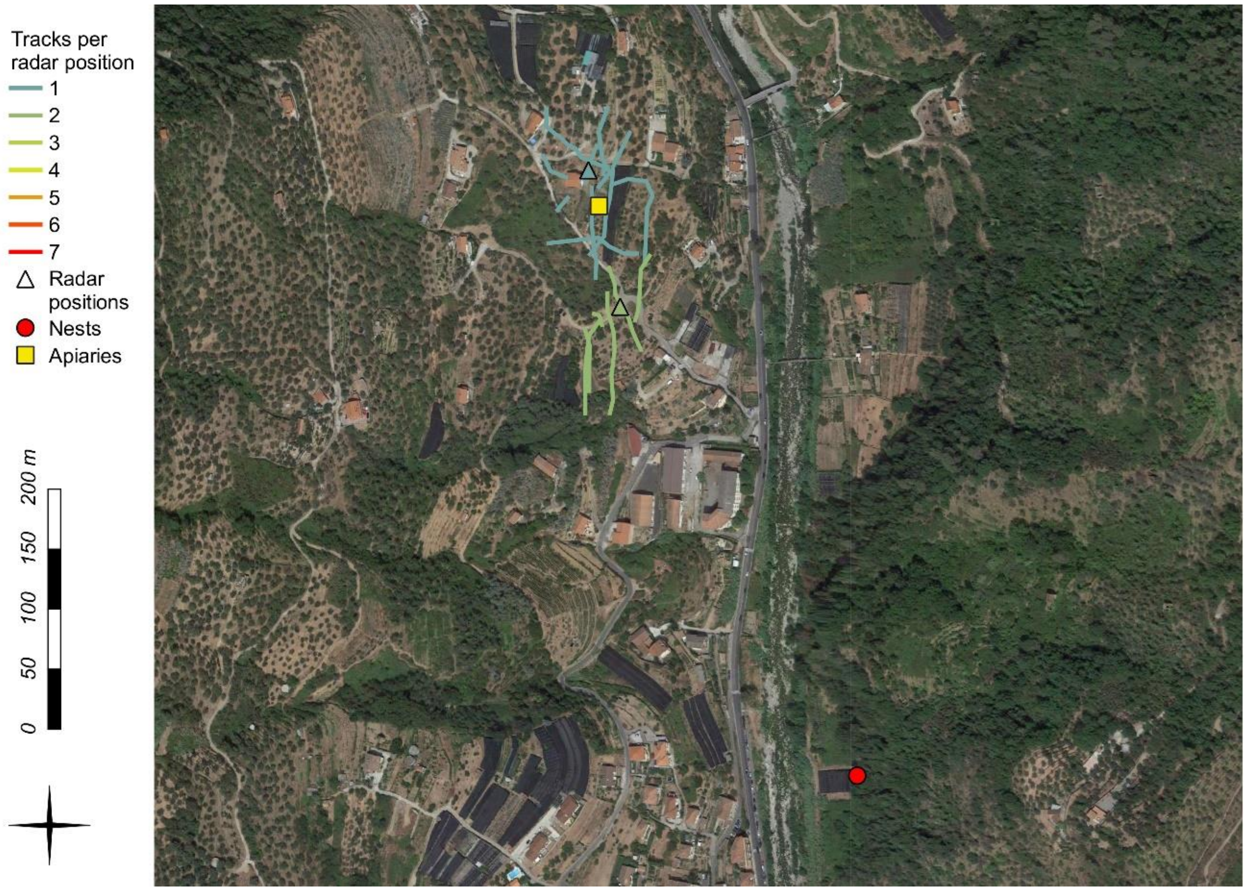

Supplementary Fig. S4 Tracking session in Dolceacqua (7-20 November 2018). Hornets were tagged in the apiary (yellow square), tracked with the harmonic radar from two positions (triangles) until nest position (red dot) was visually discovered by chance with the use of binoculars, therefore tracking session was interrupted. Background map by Google Maps (maps.google.com).

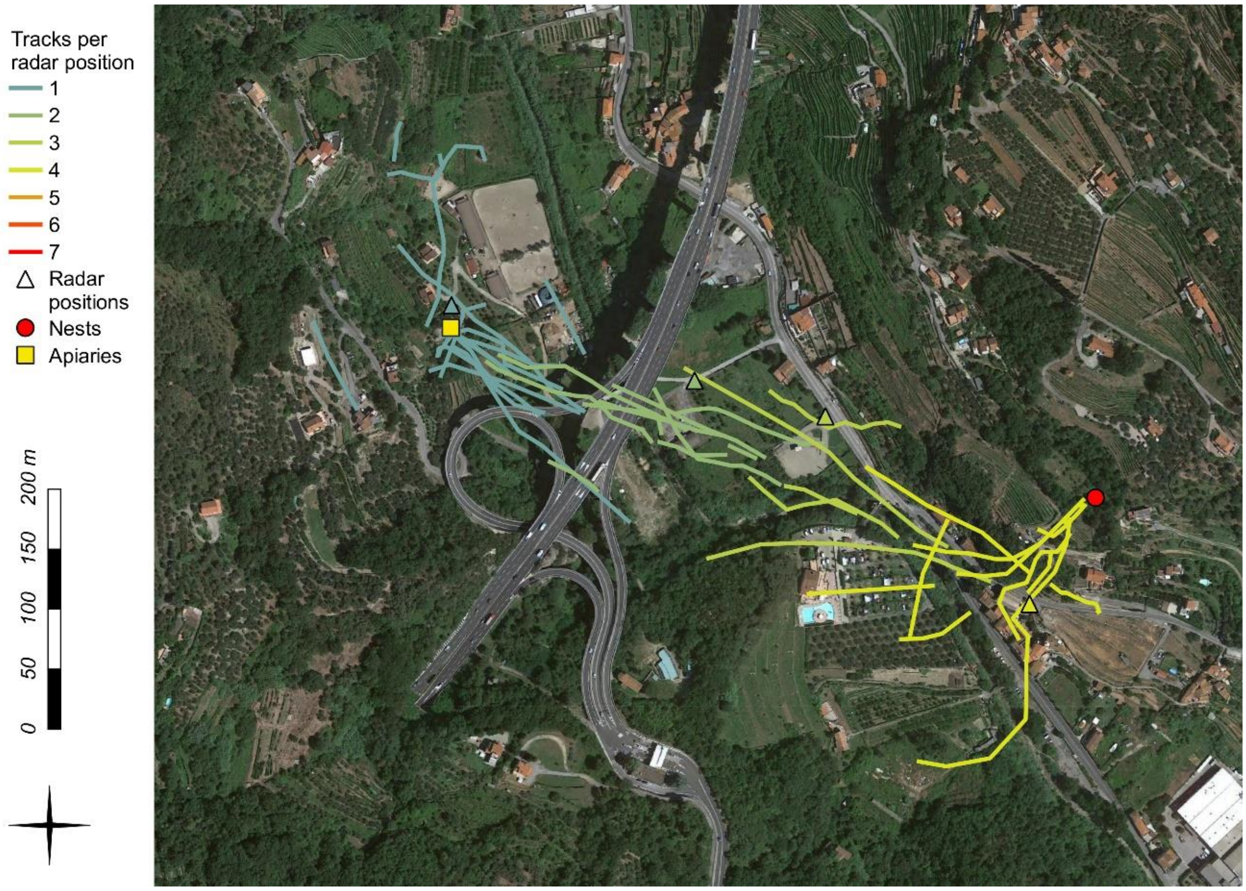

Supplementary Fig. S5 Tracking session in the outbreak of Finale Ligure (4-9 October 2018). Hornets were tagged in the apiary (yellow square), tracked with the harmonic radar from four positions (triangles) until nest position was discovered (red dot). Background map by Google Maps (maps.google.com).

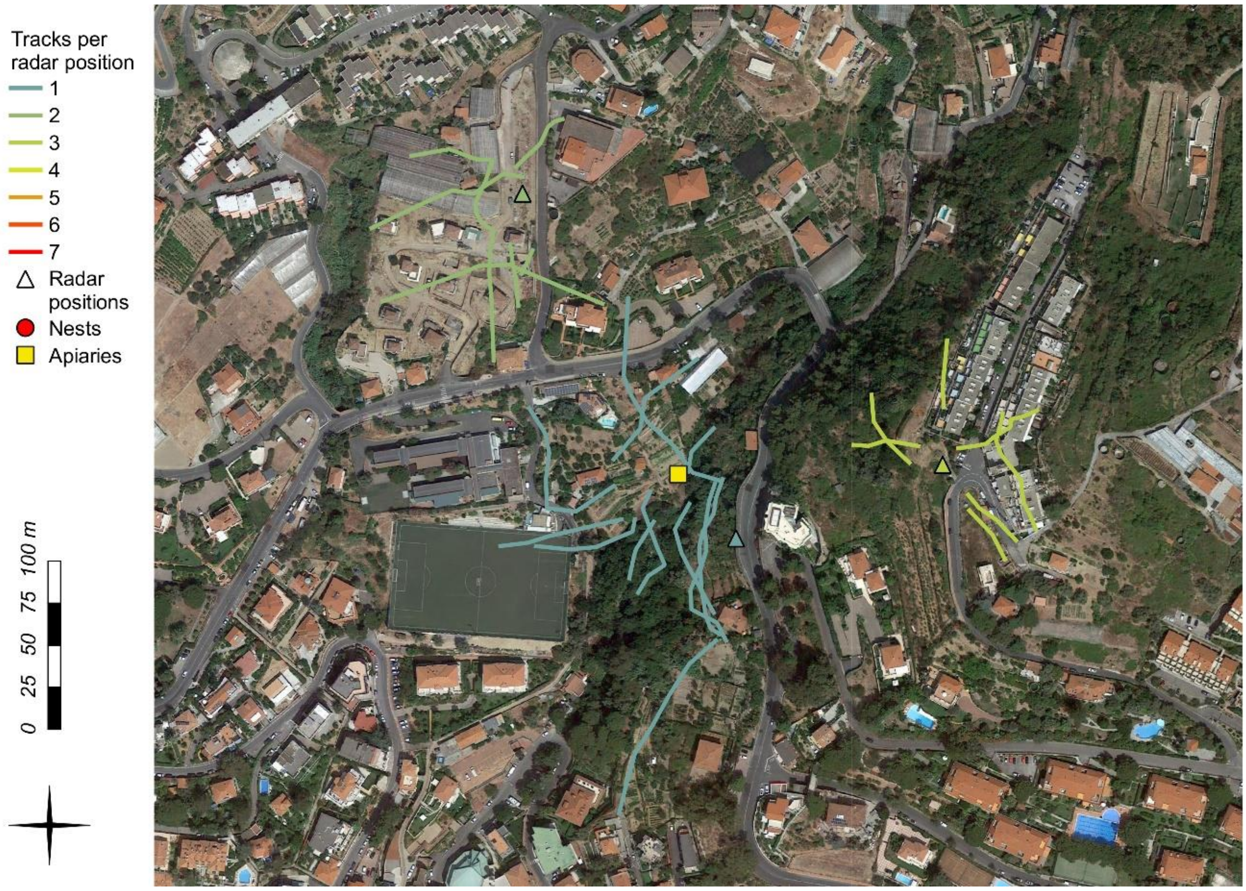

Supplementary Fig. S6 Tracking session in Ospedaletti (1-2 August 2018). Hornets were tagged in the apiary (yellow square), tracked with the harmonic radar from three positions (triangles) but nest position was not detected in this tracking session. Background map by Google Maps (maps.google.com).

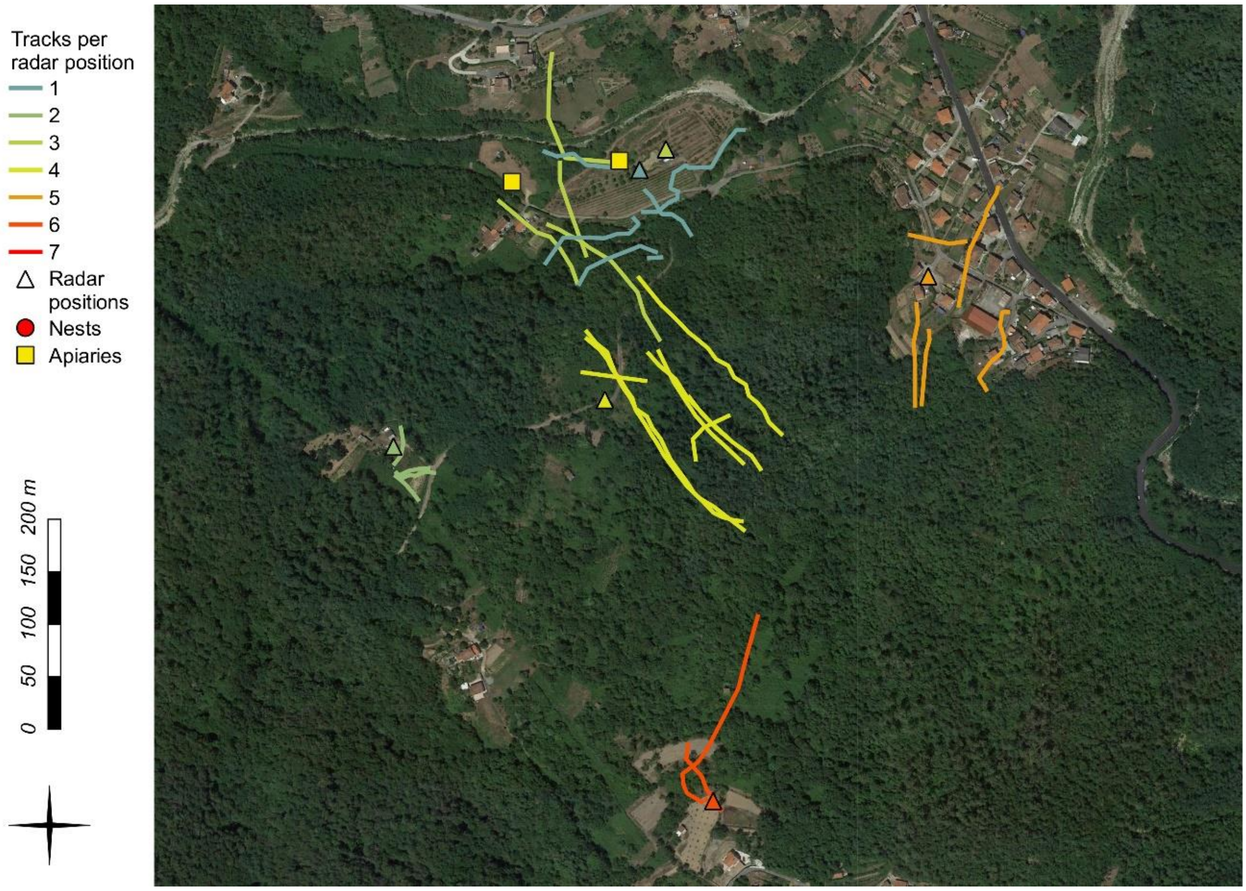

Supplementary Fig. S7 Tracking session in the outbreak of Riccò del Golfo (19 September - 3 October 2018). Hornets were tagged in the apiaries (yellow squares), tracked with the harmonic radar from six positions (triangles) but nest position was not detected in this tracking session. Tracks are pointing towards a dense vegetated area that prevented the possibility to carry on both harmonic radar tracking and on foot inspection of the area. Background map by Google Maps (maps.google.com).

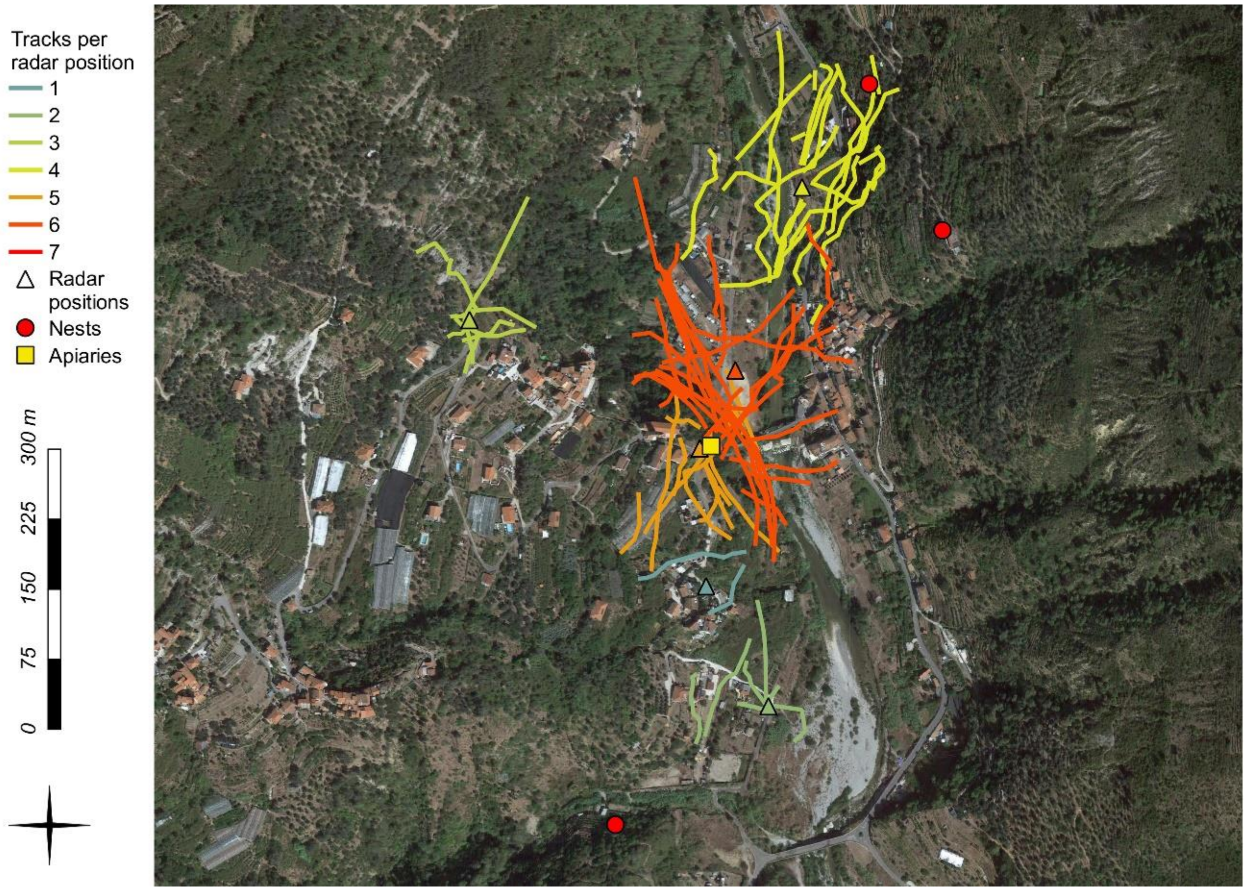

Supplementary Fig. S8 Tracking sessions in the village of Calvo of Ventimiglia in 2018 (24-27 July the first tracking session, 8-9 August the second and 12-17 October the third). Hornets were tagged in the apiary (yellow square), tracked with the harmonic radar from two positions for each tracking session (position 1-2 for the first session, 3-4 for the second and 5-6 for the third) until the position of three nests was discovered (red dots) at the end of each tracking session. Background map by Google Maps (maps.google.com).

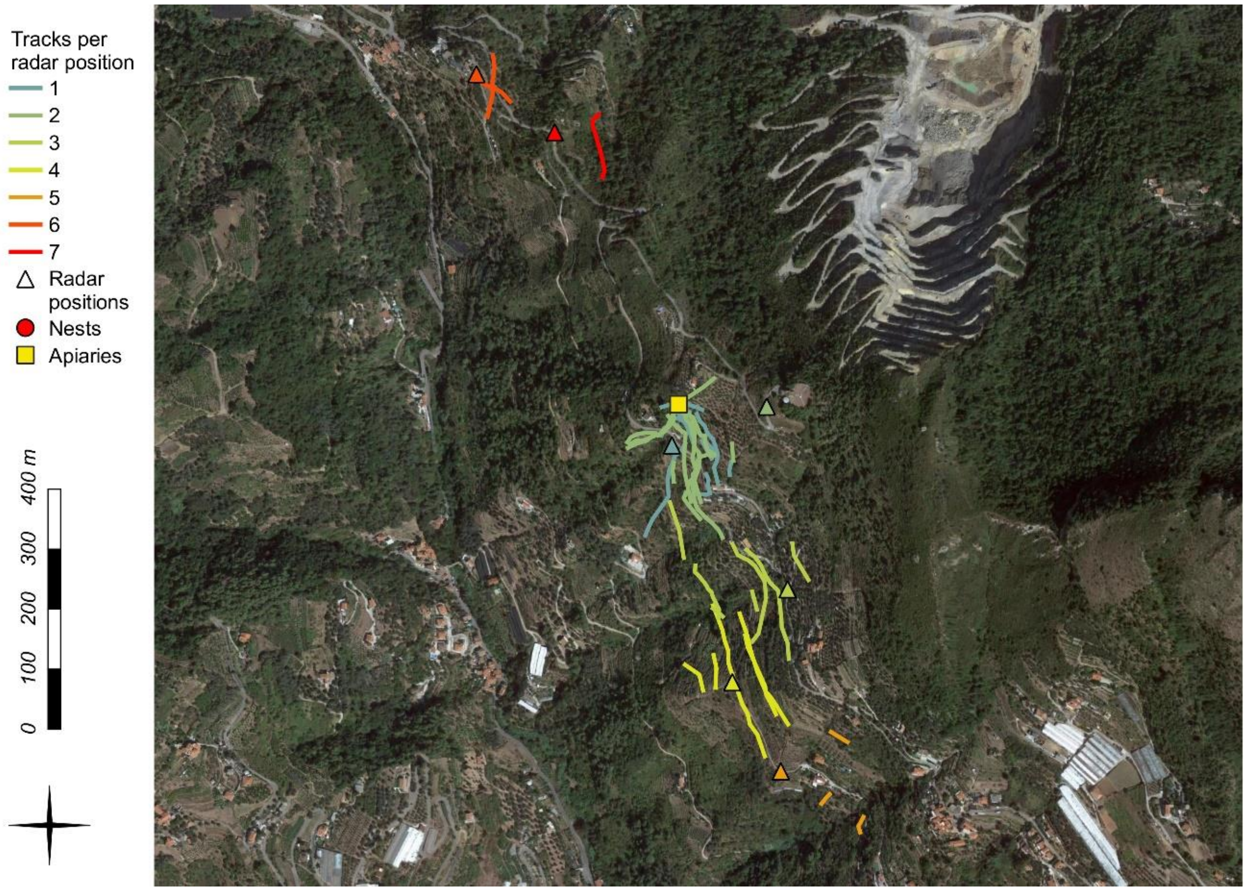

Supplementary Fig. S9 Tracking sessions in the village of Latte of Ventimiglia in 2018 (21-23 August the first tracking session, 5-6 September the second and 19-25 October the third). Hornets were tagged in the apiary (yellow square), tracked with the harmonic radar (position 1 for the first session, 2-4 for the second and 5-7 for the third) but nest position was not detected in the area. Background map by Google Maps (maps.google.com).

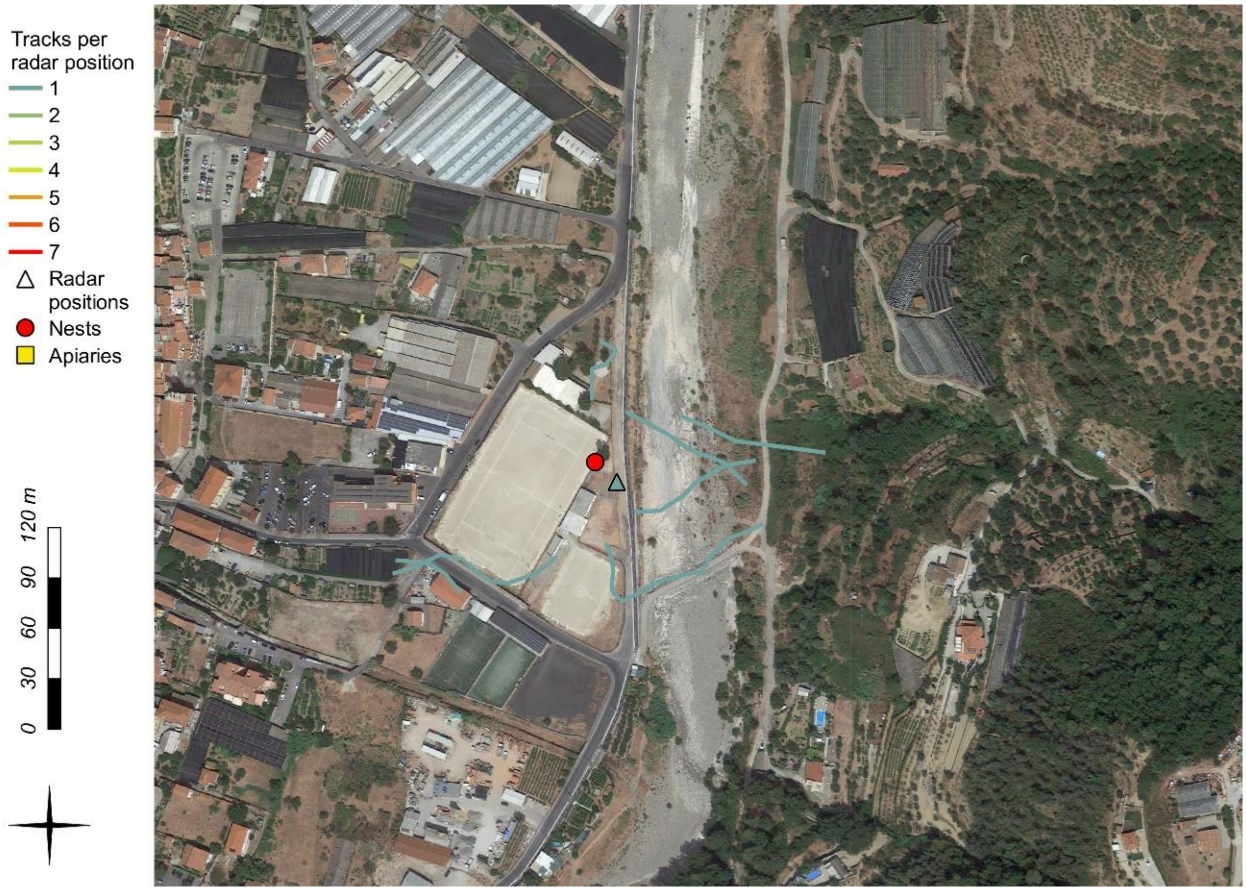

Supplementary Fig. S10 Tracking session in Camporosso (8-9 July 2019). Hornets were tagged when feeding on floral resources on the east-side of the river Nervia, tracked with the harmonic radar from one position (triangle) until nest position was discovered (red dot). Background map by Google Maps (maps.google.com).

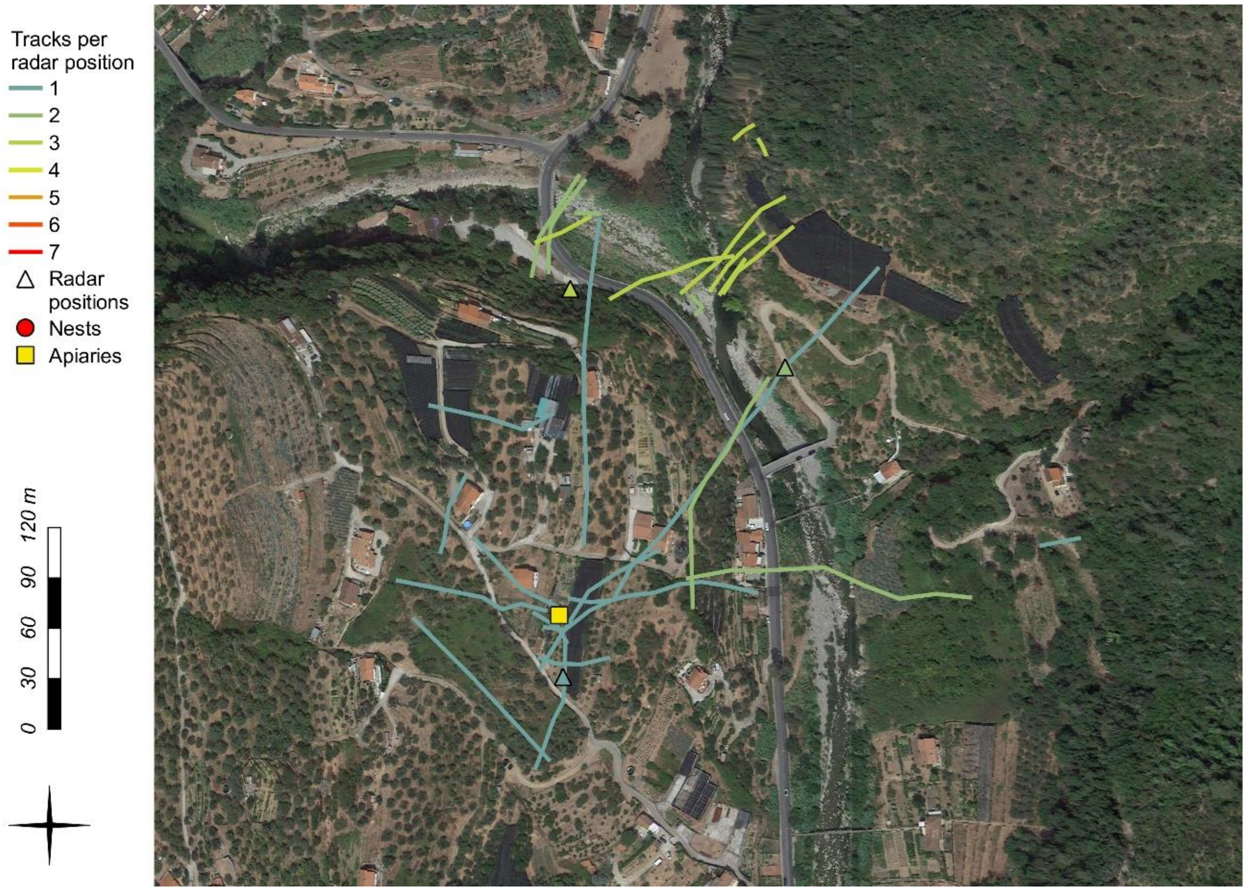

Supplementary Fig. S11 Tracking session in Dolceacqua (16-17 July 2019). Hornets were tagged in the apiary (yellow square), tracked with the harmonic radar from three positions (triangles) but nest position was not detected in this tracking session. Background map by Google Maps (maps.google.com).

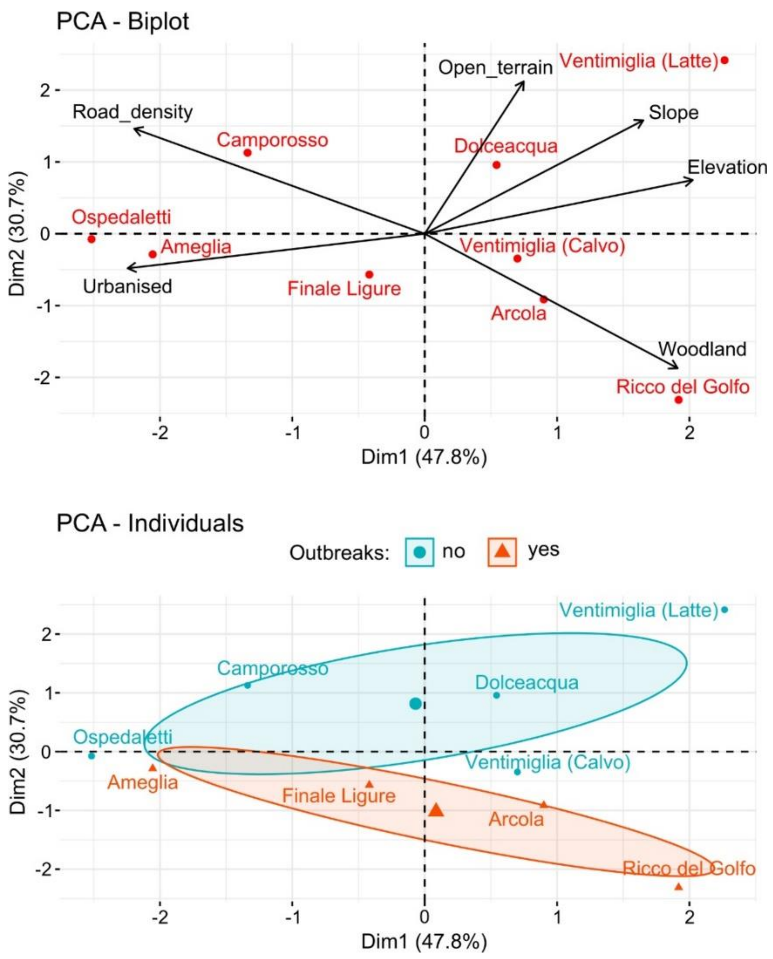

Supplementary Fig. S12 Plot of the PCA analysis on the environmental characteristics of the localities. The biplot highlights the relationship between variables and their correlation with the first and second dimension of the PCA. The individual plot divides the localities in which the harmonic radar operated between new invasive outbreaks (orange) and colonised areas (light blue); the ellipse level is set at 95%.

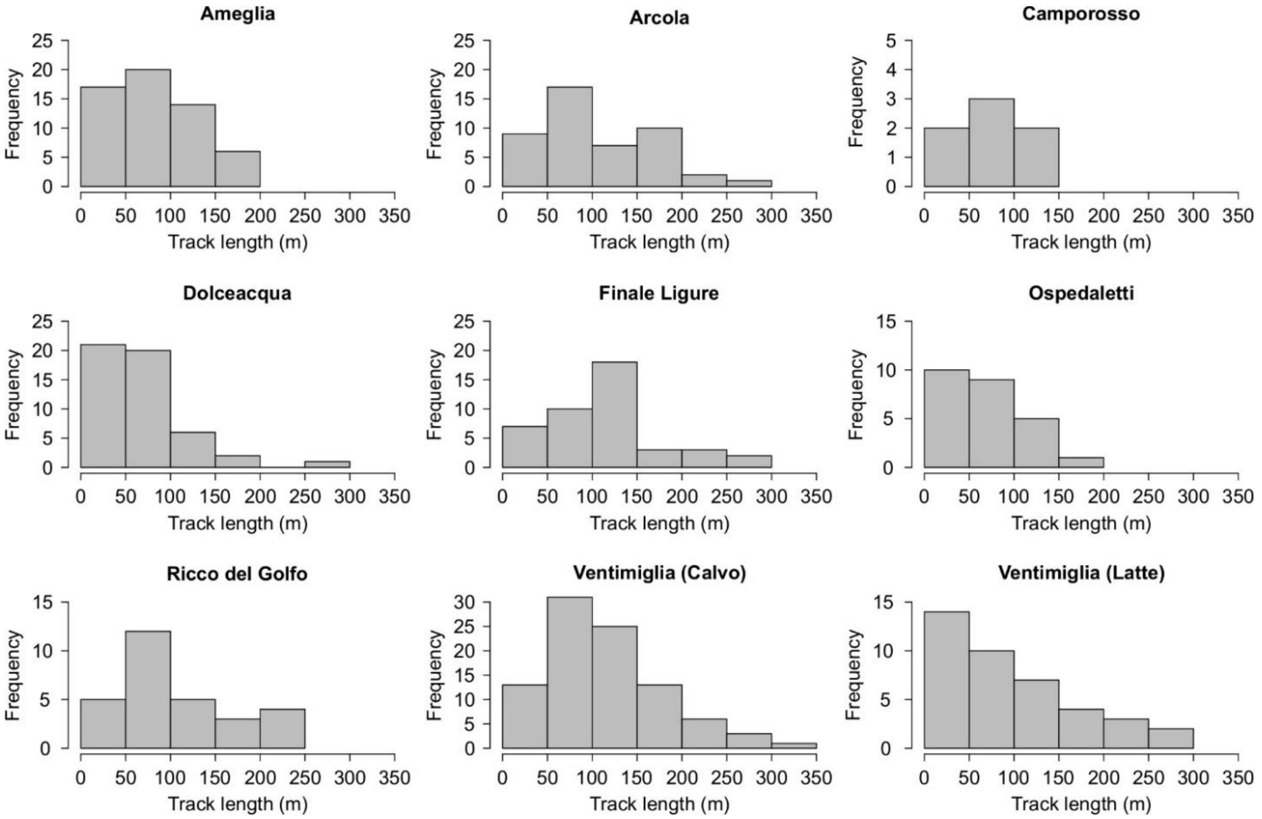

Supplementary Fig. S13 Length of the tracks of the nine localities in which the harmonic radar has operated. Sample size per locality is: Ameglia (*n* = 57), Arcola (*n* = 46), Camporosso (*n* = 7), Dolceacqua (*n* = 50), Finale Ligure (*n* = 43), Ospedaletti (*n* = 25), Riccò del Golfo (*n* = 29), Calvo of Ventimiglia (*n* = 92), Latte of Ventimiglia (*n* = 40).

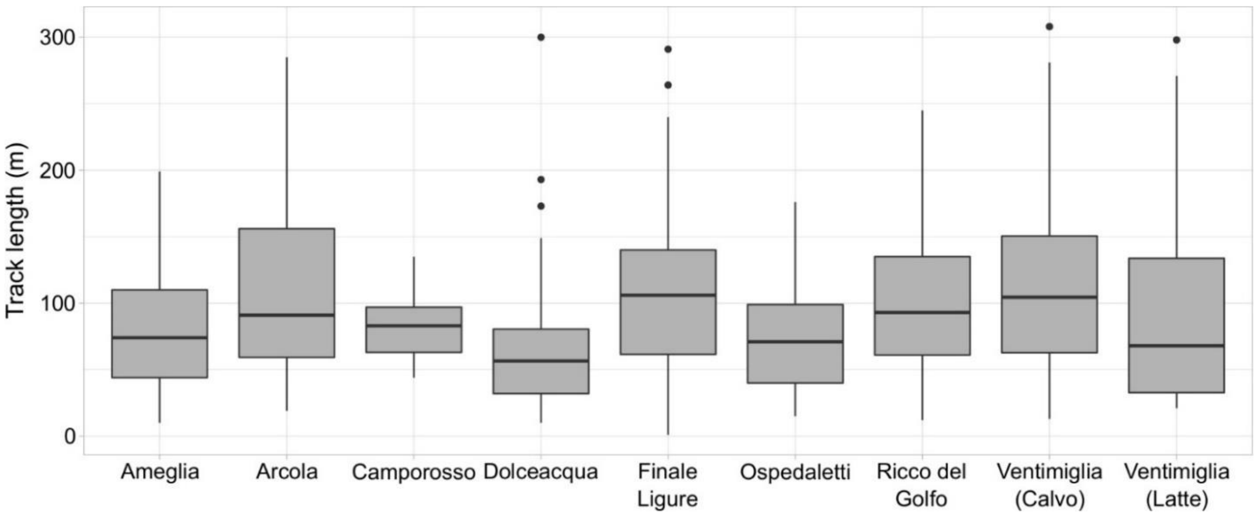

Supplementary Fig. S14 Boxplot of the length of the tracks of the nine localities in which the harmonic radar has operated. Values of the length of the tracks are similar in each locality with the exception of Dolceacqua, where tracks are shorter than the tracks in Arcola, Finale Ligure and Calvo of Ventimiglia. Sample size is the same as in Supplementary Fig. S13. Horizontal lines represent the median and points depict outlier values.

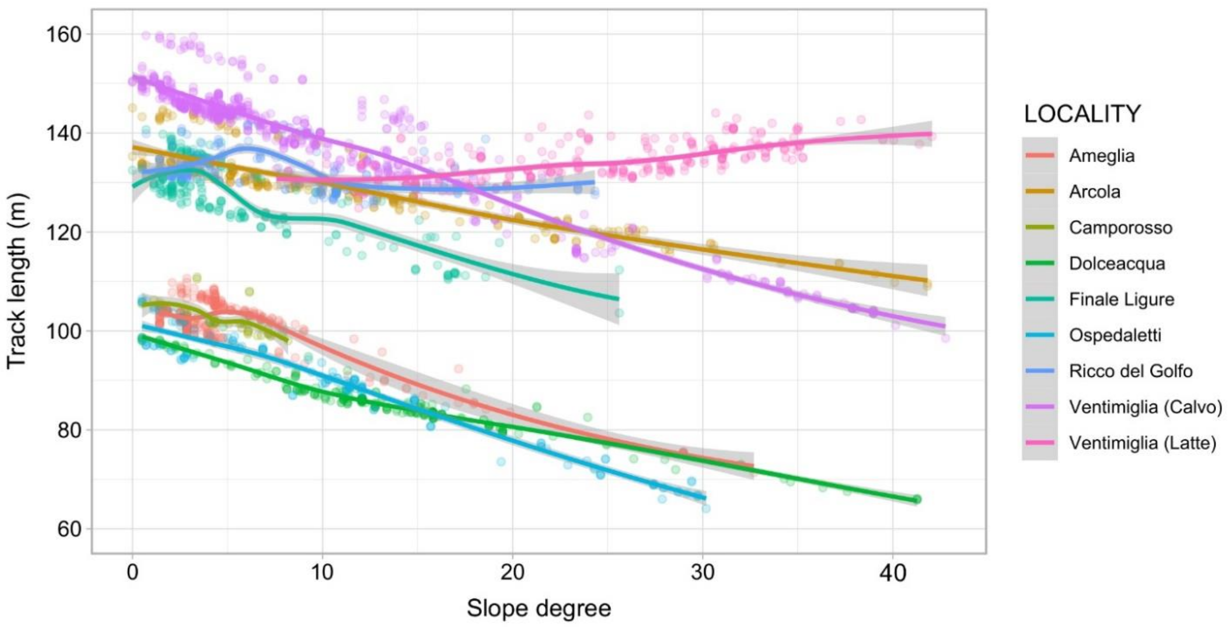

Supplementary Fig. S15 Fitted values of tracking length recorded with the harmonic radar in relation to slope degree and locality. Different colours identify the nine localities where hornets were tracked with the harmonic radar. The smoothed conditional mean was adopted for the fitting; 95% confidence intervals are displayed as grey areas.

Supplementary Table S1 Characteristics of the localities in Liguria (Italy) in which hornets have been tracked with the harmonic radar technique. The district of each locality is reported in brackets: La Spezia (SP), Savona (SV), Imperia (IM). Land cover indicates the percentage of open terrains (O), urban areas (U) and woodlands (W). Detected nests are the number of *V. velutina* nests detected with the harmonic radar tracking. For the high-density colonised areas, the range of nest density in the period 2016-2018 is reported (in these localities, the density was estimated considering the nests reported within a buffer area of 1 km). In outbreaks, the presence of a single nest prevents the possibility of providing values for nest density.

Locality	Outbreak	Nest density	Lat	Long	Land cover			Elevation		Slope		Road	Detected
		(nests km^{-1})	N	E	(%)			(m a.s.l.)		(degree)		Density	nests
					O	U	W	Mean	SD	Mean	SD	(m ha^{-1})	(*n*)
Ameglia (SP)	Yes	-	44.079	9.965	40.9	43.0	16.1	16.2	10.7	4.8	5.1	119.8	1
Arcola (SP)	Yes	-	44.104	9.913	40.7	10.3	49.0	62.7	20.8	12.9	10.4	79.5	1
Camporosso (IM)	No	2.7 - 6.7	43.814	7.632	78.8	17.0	4.3	18.3	9.4	5.8	5.1	114.9	1
Dolceacqua (IM)	No	2.0 - 2.9	43.859	7.622	66.7	5.4	27.9	93.0	23.3	14.6	10.4	105.3	2
Finale Ligure (SV)	Yes	-	44.187	8.303	43.8	27.8	28.4	47.2	16.0	8.8	7.5	89.2	1
Ospedaletti (IM)	No	4.1 - 8.4	43.805	7.719	12.5	78.7	8.8	85.0	25.9	11.9	7.6	134.1	0
Riccò del Golfo (SP)	Yes	-	44.172	9.751	23.9	11.1	65.0	139.6	27.9	8.2	5.4	49.5	0
Calvo of Ventimiglia (IM)	No	1.9 - 3.7	43.829	7.555	44.5	16.8	38.7	92.3	33.1	13.6	9.9	85.1	5
Latte of Ventimiglia (IM)	No	1.1 - 2.7	43.813	7.563	77.0	1.6	21.4	220.7	50.4	25.4	7.6	94.5	0

Supplementary Table S2 Results of the harmonic radar tracking activity. For each tracking session we report: the number of tagged hornets, the recorded number of tracks, the cumulative length of the tracks, the number of detected nests and their distance from the apiary where hornets were tagged. Furthermore, an estimate of the radar operation time and the total number of radar positions are reported.

Locality	Working days	Period from	Period to	Tagged hornets (*n*)	Tracks recorded (*n*)	Cumulative tracks length (m)	Detected nests (*n*)	Distance nest - apiary (m)	Radar operation (h)	Radar positions (*n*)
Ameglia	2	18/09/2018	19/09/2018	21	57	4630	1	786	12.0	6
Arcola	3	11/09/2018	13/09/2018	14	46	4893	1	448	23.0	7
Camporosso	2	08/07/2019	09/07/2019	6	7	582	1	136*	10.5	1
Dolceacqua	2	31/08/2017	01/09/2017	42	8	315	1	72	8.0	1
Dolceacqua	5	07/11/2018	20/11/2018	74	15	959	1	520	9.5	2
Dolceacqua	2	16/07/2019	17/07/2019	20	27	2109	0	-	14.0	3
Finale Ligure	3	04/10/2018	09/10/2018	21	43	4710	1	561	10.5	4
Ospedaletti	2	01/08/2018	02/08/2018	57	25	1840	0	-	9.3	3
Riccò del Golfo	4	19/09/2018	03/10/2018	41	29	3035	0	-	12.7	6
Calvo of Ventimiglia	2	26/10/2017	27/10/2017	47	11	1527	2	190; 197	12.5	1
Calvo of Ventimiglia	2	24/07/2018	26/07/2018	43	9	682	1	418	9.3	2
Calvo of Ventimiglia	2	08/08/2018	09/08/2018	33	32	3096	1	423	11.8	2
Calvo of Ventimiglia	2	12/10/2018	17/10/2018	48	40	5049	1	338	7.0	2
Latte of Ventimiglia	3	21/08/2018	23/08/2018	51	12	772	0	-	16.0	1
Latte of Ventimiglia	2	05/09/2018	06/09/2018	61	22	2681	0	-	15.8	3
Latte of Ventimiglia	4	19/10/2018	25/10/2018	78	6	365	0	-	8.5	3

* in the locality of Camporosso, hornets were tagged when feeding on flowers. In this case, the distance corresponds to the distance between the nest and the feeding point (this value was not included when estimating the mean distances of nests from apiaries).

Supplementary Table S3 PCA analysis on the environmental characteristics of the localities. The first table reports the contribution of the first five dimensions (Dim.1 - Dim.5) of a PCA analysis, the cumulative proportion and the respective standard deviation (SD). The second table reports the contribution of the environmental variables to the five dimensions. Biplot and individual plot of the PCA are reported in Supplementary Fig. 12.

Parameter	Dim.1	Dim.2	Dim.3	Dim.4	Dim.5
Proportion of variance	0.4776	0.3070	0.1910	0.0194	0.0050
Cumulative proportion	0.4776	0.7846	0.9756	0.9950	1.0000
Standard deviation	1.6929	1.3572	1.0705	0.3409	0.1731

Variables	Dim.1	Dim.2	Dim.3	Dim.4	Dim.5
Open terrain	2.68	33.51	26.47	2.23	0.37
Urbanised	24.10	1.72	23.82	1.53	9.31
Woodlands	17.48	26.09	0.01	10.61	20.06
Elevation	19.63	4.16	27.16	40.58	8.48
Slope	13.06	18.58	20.55	39.16	8.65
Road density	23.06	15.94	1.99	5.88	53.12

Supplementary Table S4 Results of a GLMM analysis on the relationship between the length of the tracks recorded with harmonic radar and the environmental characteristics. The model with the best fitting is a model that considers all the environmental variables as fixed effects and uncorrelated random intercepts (based on locality) with random slopes (based on slope degree of the study areas). For each variable the following is reported: the estimated coefficient (*β*), the standard error (*SE*), *Z* and *P* values. The slope degree is the main variable that negatively affects the length of the tracks, while urban areas has a positive influence on the response variable. Woodlands, elevation and road distance do not affect tracking length.

**Variables**	***β***	***SE***	***Z***	***P***
Urbanised	**0.0736**	**0.0337**	**2.182**	**0.0291**
Woodlands	0.0007	0.0331	0.022	0.9823
Slope	**-0.3325**	**0.1378**	**-2.414**	**0.0158**
Elevation	-0.1707	0.1856	-0.919	0.3579
Road distance	0.1220	0.1194	1.022	0.3068

Selected model: Track_lenght ~ Landcover + Slope + Elevation + Road_distance + (Slope || Locality)
Comparison between the selected model (AIC -1013) and the null model (-981): $\chi^2 = 44.19$, $df = 6$, $P < 0.001$
